# Diagnosis and Treatment of Hypophosphatasia

**DOI:** 10.1007/s00223-025-01356-y

**Published:** 2025-03-06

**Authors:** L. Seefried, F. Genest, C. Hofmann, M. L. Brandi, E. Rush

**Affiliations:** 1 Osteology and Clinical Trial Unit, König-Ludwig-Haus, University of Würzburg, Würzburg, Germany; 2 Pediatric Rheumatology and Osteology, University Children’s Hospital Wuerzburg, Würzburg, Germany; 3 F.I.R.M.O. Italian Foundation for the Research on Bone Diseases, Florence, Italy; 4 Division of Clinical Genetics, Children’s Mercy Kansas City, Kansas City, MO USA; 5 Department of Pediatrics, University of Missouri – Kansas City School of Medicine, Kansas City, MO USA; 6 Department of Internal Medicine, University of Kansas School of Medicine, Kansas City, KS USA

**Keywords:** Hypophosphatasia, Alkaline phosphatase, Phenotypes, Subclinical, Canonical, Non-canonical, Diagnostic criteria, Treatment

## Abstract

Hypophosphatasia (HPP) is a rare inherited metabolic disorder characterized by deficient activity of tissue-nonspecific alkaline phosphatase (TNAP) caused by variants in the *ALPL* gene. Disease manifestations encompass skeletal hypomineralization with rickets and lung hypoplasia, vitamin B6-dependent seizures, craniosynostosis, and premature loss of deciduous teeth. The clinical presentation can comprise failure to thrive with muscular hypotonia, delayed motor development, and gait disturbances later in childhood. In adults, pseudofractures are a characteristic indicator of severely compromised enzyme activity, but non-canonical symptoms like generalized musculoskeletal pain, weakness, and fatigue, frequently accompanied by neuropsychiatric and gastrointestinal issues are increasingly recognized as key findings in patients with HPP. The diagnosis is based on clinical manifestations in combination with persistently low alkaline phosphatase (ALP) activity, elevated levels of ALP substrates, specifically inorganic pyrophosphate (PPi), pyridoxal 5'-phosphate (PLP) or urine phosphoethanolamine (PEA), and genetic confirmation of a causative *ALPL* variant. Considering the wide range of manifestations, treatment must be multimodal and tailored to individual needs. The multidisciplinary team for comprehensive management of HPP patients should include expertise to ensure disease state metabolic and musculoskeletal treatment, dental care, neurological and neurosurgical surveillance, pain management, physical therapy, and psychological care. Asfotase alfa as first-in-class enzyme replacement therapy (ERT) for HPP has been shown to improve survival, rickets, and functional outcomes in severely affected children, but further research is needed to refine how enzyme replacement can also address emerging manifestations of the disease. Prospectively, further elucidating the pathophysiology behind the diverse clinical manifestations of HPP is instrumental for improving diagnostic concepts, establishing novel means for substituting enzyme activity, and developing integrative, multimodal care.

## Background

Hypophosphatasia (HPP) is a rare genetic disorder due to deactivating variants in the *ALPL* gene (Chr 1p36.12, OMIM 171760) leading to deficient activity of tissue-nonspecific alkaline phosphatase (TNAP). Accordingly, confirmation of the underlying genetic variant in combination with an increase of the direct ALP substrates inorganic pyrophosphate (PPi) and pyridoxal-phosphate (PLP) as well as a related increase in phosphoethanolamine (PEA) are important laboratory features for diagnosis of the disorder [1–3].

Over the last decade, improved awareness for the disease has broadened our perspective on the underlying pathophysiology and associated clinical manifestations. Building on the nosology of different phenotypes according to age of disease onset, [4, 5] for obvious reasons, there has always been a strong clinical and scientific focus on pediatric-onset phenotypes and skeletal manifestations. Still, growing evidence supports an expanded understanding of the clinical significance of the disease across lifetime and specifically the wide range of non-canonical manifestations particularly in adult patients.

### Pathophysiology

The pathophysiology of HPP is intricate and more complex than commonly reflected in current literature, at least when it comes to providing molecular explanations for the wider range for clinical signs and symptoms and substantiating the causality in adults with concomitant, aging-related conditions. The essential concept of pathogenic ALPL variants causing deficient enzyme activity with the consequence of reduced breakdown of the mineralization inhibitor PPi is well understood and explains many of the canonical clinical presentations of the disease, specifically rickets and osteomalacia with resulting deformities as well as pseudofractures [1, 6]. Similarly, enhanced formation of calcium pyrophosphate dihydrate (CPPD) crystals triggering inflammatory joint and tissue responses in HPP appears conclusive [7]. Furthermore, the pathophysiology behind early loss of deciduous teeth has been associated with elevated PPi levels, which appears reasonable considering impaired mineralization of dentin matrix and hypoplasia of the acellular extrinsic fiber cementum of exfoliated teeth [8]. Further clinical manifestations are hypothesized to occur due to compromised vitamin B6 turnover and deficient intracellular and neuronal availability of vitamin B6 [9]. However, notwithstanding the well-established role of vitamin B6 as an important cofactor for numerous enzymes involved in the turnover of amino acids and neurotransmitters, the clinical implications in terms of neuropsychiatric manifestations of the disease are incompletely understood and not clearly defined [3, 10, 11]. Eventually, we anticipate more robust evidence to unanimously assign reported neuropsychiatric manifestations like neuropathic pain, dysaesthesia, headache, migraine, anxiety, depression, and mental fatigue [12–15] to one or more of multiple hypothesized mechanisms such as axonal development, GABA synthesis, serotonin/dopamine conversion requiring PLP-dependent aromatic amino acid decarboxylase (AADC), or other as yet unknown mechanisms [16]. While it may also appear reasonable to hypothesize a pathophysiologic association of altered and deficient conversion of pro-inflammatory ATP to anti-inflammatory adenosine underlying systemic musculoskeletal pain, the clinical significance of this concept has not been confirmed in HPP patients [11, 17].

Similarly, gastrointestinal symptoms with nausea, vomiting, and feeding difficulties in infancy and reported diverse aspects of gastrointestinal dysfunction in adults are well recognized but the hypothetical role of tissue-nonspecific alkaline phosphatase in the detoxification of bacterial lipopolysaccharides or other impacts on the intestinal microbiome or the absorption of phosphate-containing lipids have not been substantiated in HPP patients [12, 18]. Still, expression of the tissue-nonspecific isoform ALPL has been described in human gut mucosa and literature suggests that ALPL contributes to intestinal dephosphorylation processes [19]. Further intestinal ALP (ALPI) and ALPL may to some extent compensate for one another in case one is deficient [20]. Parenthetically, one might also speculate that deficient ALP activity may interfere with energy supply in working muscles or that enzyme deficiency in working muscles can lead to compromised degradation of metabolic end products which might accumulate during exercise or labor and could potentially explain both, limited endurance capacity as well prolonged recovery after exertion [21, 22]. Still, the underlying molecular details are not yet appropriately clarified.

### Genetics

With over 400 defined variants reported for the *ALPL* gene and with severely affected patients typically harboring biallelic variants either in homozygous or various compound heterozygous combinations, the genetic background of HPP and the specific impact of individual variants have been challenging to completely understand. While efforts are undertaken to reclassify variants of uncertain significance, it becomes clear that there exists a broad individuality of clinical manifestations, even in patients with the same genotypes, largely precluding predictive statements on a clinical phenotype [2]. Inheritance of HPP is regularly described to follow either an autosomal recessive or autosomal dominant pattern with biallelic variants generally causing a more severe phenotype, while dominant negative effects are supposed to explain clinical manifestations in heterozygous patients [23]. While this simplified concept offers a rudimentary understanding, it is surely insufficient to explain the wide spectrum of individual clinical presentations and severity and the clinical course of the disease across the lifespan.

Accordingly, it appears reasonable to assume that further mechanisms like loss of heterozygosity, haploinsufficiency, gene silencing/imprinting, and further modifiers of expressivity and penetrance might play a role. In that regard, the impact of regulatory domains and putative deep intronic variants which might affect splicing or which might function as quantitative trait loci should be of particular interest. Further, the roles of specific haplotypes and distinct genes, functioning as epigenetic modifiers or interfering with post-transcriptional and post-translational processing and function of the enzyme have to be researched.

Further, the impact of tissue-specific, age-related, and environmental factors on the regulation of expression and post-transcriptional processing of the enzyme requires further attention and along with the molecular modifiers that convey such signals. Post-transcriptional and post-translational mechanisms are already known to affect enzyme activity in a tissue-specific manner by means of glycosylation patterns and these may also influence secondary and tertiary structure and function of the enzyme [24].

Accordingly, better understanding the genomic and epigenetic landscape and associating clinical manifestations with pathophysiological mechanisms will be crucial not only to better understand genotype–phenotype correlations but more importantly, to better understand the course of disease manifestations across lifetime and to further refine diagnostic approaches and enable individually optimized treatment and tailored interventions in the future [25, 26].

## Clinical Manifestation

Research in HPP has—for obvious reasons—primarily focused on severe childhood manifestations. However, increasing data are shedding light on the significant symptomatology and disease manifestations experienced by adults with HPP, even in the absence of osteomalacia or an otherwise discernible bone phenotype [28]. In contrast to what has canonically been reported and documented, adults with HPP not only suffer from consequences of bone and joint manifestations and compromised bone mineralization but rather from extra-skeletal reduced physical performance, increased fatigue, and musculoskeletal pain [27, 29, 30]. Additionally, patient-reported outcomes further highlighted the profound impact of HPP on quality of life and physical function, irrespective of disease age of onset, emphasizing the importance of addressing their substantial disease burden [27].

### Operational Diagnostic Criteria for Children and Adults

Even though it may appear straight forward at first glance, diagnosing patients with HPP can be challenging in clinical practice for both, pediatric and adult populations [31, 32]. On the one hand, this is surely owing to the wide range of relatively nonspecific clinical manifestations but on the other hand this is also related to the long-standing lack of clear-cut operational diagnostic criteria. Insights from the global HPP registry substantiated the well-known diagnostic delay of the disease not at least due to the wide spectrum of clinical manifestations across all age groups [33]. To harmonize diagnostic approaches to HPP, to facilitate patient identification, and to increase diagnostic accuracy, a series of three joint publications from the HPP international working group (IWG) assessed the multifaceted clinical presentations and diagnostic hallmarks of HPP, shedding light on the diagnostic value of specific signs and symptoms of the disease to eventually propose consensus diagnostic criteria for children and adults. While one of these manuscripts provides an overview of the methodological approach [34], the other two specifically address diagnostic pathways for children [35] and adults [36].

**Fig. 1:**
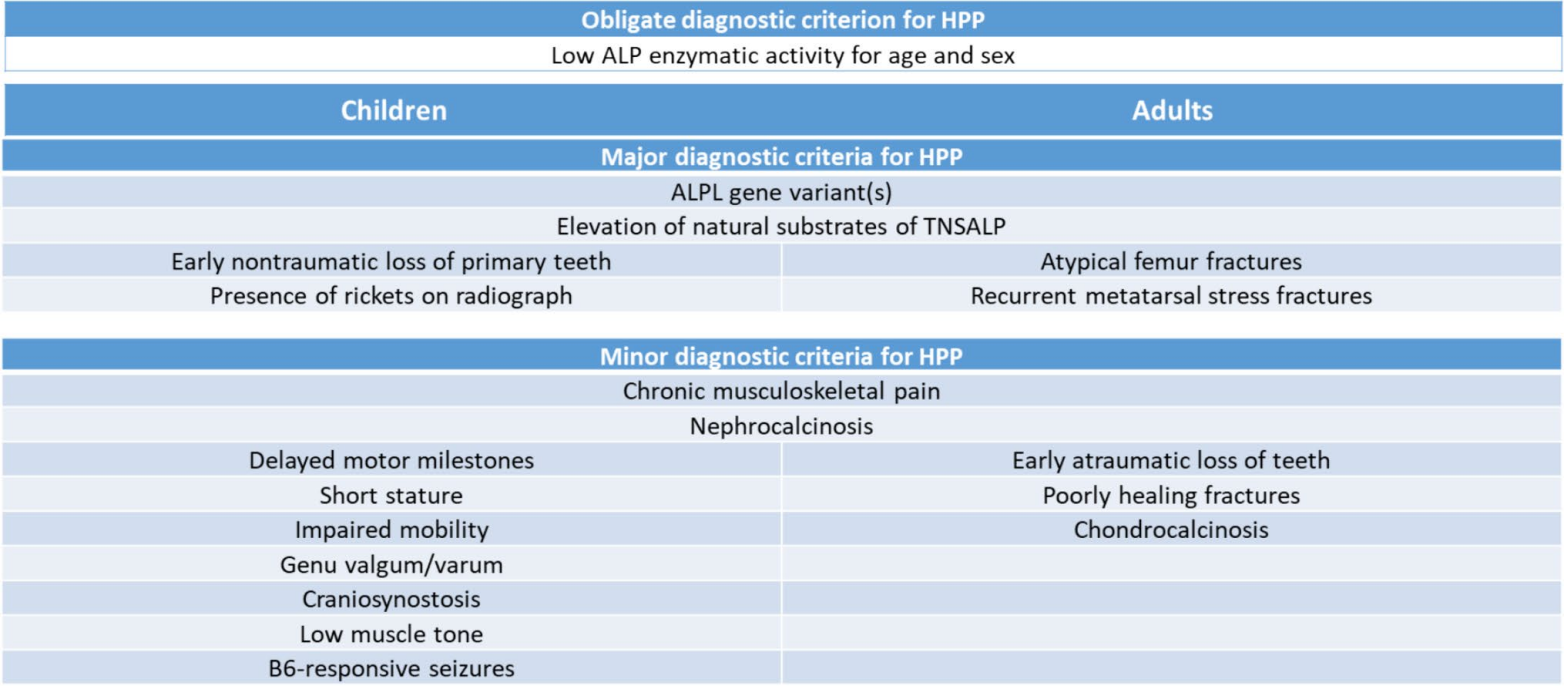
Currently recommended diagnostic criteria for HPP in children (left column) and adults (rights column). HPP 2 major criteria or 1 major and 2 minor criteria. Low ALP activity is an obligate criterion and requires exclusion of other conditions that can cause low ALP activity and repeated measures to avoid misinterpretation of situational reductions. HPP, hypophosphatasia; TNSALP, tissue-nonspecific alkaline phosphatase (According to Rush et al., *Osteoporos Int.* 2023, Brandi ML, et al. *Osteoporos Int.* 2023; Khan AA, et al. *Osteoporosis Int*. 2023)

As an overarching principle, it was agreed that beyond the mandatory requirement of reduced ALP activity, patients must meet 2 major or 1 major and 2 minor criteria in order to make a clinical diagnosis of HPP. While clinical criteria were defined separately for children and adults, confirmation of a genetic variant and elevated substrate levels are considered major criteria in both groups. Focusing further on the diagnostic hurdles encountered in pediatric HPP, a meticulous analysis of existing literature by the IWG identified 15 clinical and laboratory indicators that appeared to have a pivotal role in HPP diagnosis in children [35]. Additional major criteria include premature tooth loss and radiographically confirmed rickets. Other classical manifestations including craniosynostosis, vitamin B6-dependent seizures, delayed development, impaired mobility, and reduced muscle tone were considered minor criteria [31]. In adults, the corresponding body of literature findings reflects the current, transitional state and ongoing learning in the perspective on HPP [36]. Accordingly, the 17 diagnostic indicators identified covered a very diverse array of clinical phenotypes related to the condition and include chondrocalcinosis, poorly healing fractures, early atraumatic loss of teeth, nephrocalcinosis, and chronic musculoskeletal pain as minor criteria. Recurrent metatarsal fractures and the both well-established and characteristic finding of pseudofractures were assigned major criteria. A summary of currently proposed diagnostic criteria is provided in Fig. 1.

### Circulating Alkaline Phosphatase

As an overarching principle for the publication of diagnostic criteria, it was agreed among the international working group that following current evidence, reduced circulating ALP activity below the age- and sex-specific reference range was mandatory to further consider a diagnosis of HPP. In that regard, primarily assessing total ALP activity as an initial assessment is appropriate and sufficient to identify putative HPP patients since the disease-causing ALPL gene constitutes the genetic background for all bone and liver isoforms of the enzyme, which normally account for > 90% of circulating ALP activity [1, 37]. However, in specific situations such as third trimester of pregnancy, certain tumors, inflammatory bowel disease, or other conditions potentially affecting the integrity of cells and tissues expressing genetically distinct alkaline phosphatases, these may substantially contribute to total serum/plasma ALP activity. In such situations, differential analysis of ALP isoenzymes can provide supportive information [38]. Hence, even though exceptions may apply and constellations with situationally normal or technically low normal but relatively too low ALP activity are conceivable, this mandatory key criterion constitutes an important starting point for generally applicable, harmonized diagnostic criteria [38, 39]. Taking into account the complex regulation of ALPL expression and activity, it appears equally important to consistently rule out potential other causes for low circulating ALP activity due to other conditions or preanalytical flaws like hemolysis or sampling in EDTA tubes. In that regard, it is also important to understand that ALP activity in serum or plasma alone does not correlate immediately with clinical disease severity nor does it accurately reflect actual enzyme function within different tissues.

The spectrum of differential diagnoses for low AP in adulthood is broad, encompassing various metabolic conditions and chronic diseases. In clinical practice, specific attention should be given to ongoing or recent antiresorptive therapies such as bisphosphonates, denosumab or hormonal treatments such as estrogen, which also may reduce ALP levels. Lists of further conditions that can inconsistently be associated with reduced circulating ALP activity have been published elsewhere [3].

### Elevated ALP Substrates Levels

In the setting of reduced ALP levels, confirmation of elevated ALP substrates is considered a conclusive laboratory finding and a major diagnostic criterion, underscoring the pathophysiological significance of deficient enzyme activity.

While considered the most sensitive and specific substrate for diagnosing HPP and the major cause for compromised mineralization as a hallmark of the disease [40], assessment of plasma PPi is not readily available in routine clinical use at this time. The main reason for this unfortunate situation is the very elaborate and complex preanalytical procedure to obtain valid, unadulterated samples not biased by artifacts. In addition, several technical methods for quantitation of PPi have been described by different labs and finding the best option and making results comparable is still subject to further optimization among the laboratories who have established this analysis [41, 42]. Accordingly, while PPi testing is currently restricted to expert centers and clinical trials, it may become generally available for routine assessments in the future.

Pyrodoxal-5-phosphate (PLP) is the main circulating form of vitamin B6, although it is important to understand that all three vitameric forms (pyridoxal (PL), pyridoxamine (PM), and pyridoxine (PN)) can be phosphorylated to PLP, PMP, and PNP, respectively. Eventually, all of them are degraded to 4-pyridoxic acid (4-PA) which is then excreted in the urine. In the setting of deficient ALP activity, both intestinal dephosphorylation of Vitamin B6 from nutrients as well as dephosphorylation of PLP in other tissues are compromised, leading not only to deficient intestinal absorption of vitamin B6 but also an accumulation of circulating PLP and an imbalance in the ratio of PLP to PL and/or pyridoxic acid (PA). This correlation has been evaluated as a means to assess disease severity but statistical analysis of eventually revealed insufficient precision so that the findings could not be used to assess disease severity [43]. Since PLP and these conversions are photosensitive [44], samples in clinical practice should be protected from light exposure to avoid premature degradation and shift in the proportional content.

Elevated levels of PEA can be found in both blood and urine of HPP patients but it seems that results from urine, normalized to urinary creatinine provide most reliable results to serve as a useful assay in clinical practice, although the rate of normal results in less severely affected patients is high. This may be due to the fact that PEA is not a direct ALP substrate and its elevation rather an indirect consequence which may result from the breakdown of the glycosylphosphatidylinositol (GPI) anchor of membrane-associated proteins like TNAP or potentially deficient hepatic processing of PEA by PLP-dependent enzymes [10].

There are most likely further substrates and metabolites that are materially important and may pathophysiologically explain the wide range of clinical manifestation in HPP. These include the cascade of ATP processing to ADP/AMP and Adenosine with a resulting imbalance between pro-inflammatory ATP and deficient anti-inflammatory Adenosine. Considering the above compounds are key ligands with distinct affinity to different purinoceptors, their ALP-regulated proportional availability presumptively also interferes with further purinergic signaling mechanisms and clinical manifestations beyond inflammation [45]. Similarly, there have been reports connecting ALP activity with provision of (phospho-)osteopontin from animal studies and it may appear conclusive that this could also involve other small integrin-binding ligand, N-linked glycoproteins (SIBLING) but much remains to be done until such hypothetical concepts can be harnessed for diagnostic purposes. Unfortunately, the pathophysiological role of these aforementioned compounds in the clinical context of HPP has not been evaluated to date and they are not available for routine clinical testing.

Further, the way how patients describe the nature of their pain suggests that deficient ALP activity might cause delayed procurement of pro-inflammatory, pain-associated substrates occurring during physical activity. Their progressive accumulation and protracted degradation of such compound during exercise could then explain both, earlier fatigue during physical activity, as well as prolonged regeneration.

### Genetic Testing

Confirmation of an ALPL variant should be considered standard of care and whenever feasible, this should be accomplished early during the diagnostic workup for HPP [38] to avoid misdiagnosis and inappropriate treatment. While the finding of a pathogenic or likely pathogenic is considered a major criterion, it is not an obligate criterion, i.e., a diagnosis of HPP can be made without having the results of genetic testing for several reasons. First, genetic diagnostics takes time and should not delay timely and life-saving treatment in severely affected infants. Second, genetic testing is not readily available in all regions of the world. Third, like nearly all genetic disorders, the diagnosis yield of genetic testing is not 100%, with a small but non-trivial burden of findings of variant negative disease or variants of uncertain significance. Efforts are underway to further reduce both and provide more clarity for these patients. [26, 46]. In that regard, it is important that clinicians provide accurate, meaningful information to assess “variants of unknown significance,” both for the individual case and also to support respective efforts by the Global ALPL gene variant classification project [26].

### Diagnostic Perspective

The diagnostic hallmarks of the most severe cases of HPP with potentially life-threatening manifestation in the perinatal setting are clinically unambiguous and should not create a diagnostic dilemma [47]. These patients are severely compromised with almost absent mineralization of the skeleton, respiratory failure, thoracodystrophy, lung hypoplasia, and vitamin B6-dependent seizures [32, 48, 49]. Prompt diagnosis and multidisciplinary management are essential to prevent potentially lethal complications. Additionally, patients may develop craniosynostosis, failure to thrive, and an array of gastrointestinal issues including frequent nausea and vomiting during infancy and early childhood [3]. Premature loss of deciduous teeth before the age of 5 years is another canonical indicator of HPP. Less specific but still very characteristic symptoms during childhood include muscular hypotonia, delayed motor development, and a quite characteristic waddling gait disturbance. During late puberty and in early adulthood, many patients experience an improvement in their perceived burden of disease and can manage their condition quite well [3]. With progressive aging, many of these patients with canonical childhood manifestations will also experience reemergence of the disease in adulthood. The most specific adult manifestations in these patients are pseudofractures, i.e., persistent or progressive focal demineralization of bones causing persistent or recurrent pain upon loading due to prolonged mineralization issues [50]. In some instances, pseudofractures may eventually progress to complete discontinuity. Although pseudofractures have been described at many skeletal sites in HPP, they most frequently become symptomatic in the load-bearing lower extremities, specifically the femur and metatarsal bones [27, 48, 51]. In that regard, adult patients with pseudofractures should be evaluated for a potentially missed medical history of canonical childhood manifestations like rickets or craniosynostosis for a comprehensive understanding of the individual disease manifestation [52].

On the other end of the wide spectrum of HPP disease burden, many patients with meaningful residual enzyme activity display an apparently normal skeletal development and mineralization and do not experience clinical disease manifestations during childhood and early adulthood. Traditionally, this has been described as “adult-onset” disease, although enzyme activity in these patients has likely been compromised their entire lives and identification of emerging disease activity is not least a matter of how medical history is obtained and how well we know what to ask for [5]. Still, we have to acknowledge that there is a large proportion of people with a genetic variant in the ALPL gene and the biochemical signature of HPP who do not exhibit clinically relevant symptoms, and thus may be considered subclinical [52]. For yet undefined reasons, many of them eventually become symptomatic during adulthood, frequently after 40 years of age, even though this too can vary. Conversely, considering the high prevalence of heterozygosity for variants in the *ALPL* gene in the general population [53], a large proportion of people even with a pathogenic or likely pathogenic *ALPL* variant never experience clinical manifestations and may or may not have biochemical hallmarks of the disease. Those who become symptomatic only in adulthood in most instances exhibit non-canonical manifestations of the disease. This includes generalized musculoskeletal pain, weakness, poor endurance, fatigue, slow recovery from exertion, and even less specific symptoms like headache/migraine, neuropathic pain, brain fog, and nonspecific gastrointestinal and dental/periodontal issues [54–56]. The lack of specificity of many of these findings has created challenges in attaching them to the broader patterns of the disease. Still, the coincidence of several of these manifestations combined with a recognized genetic and biochemical signature appear conclusive to substantiate the diagnoses.

**Fig. 2:**
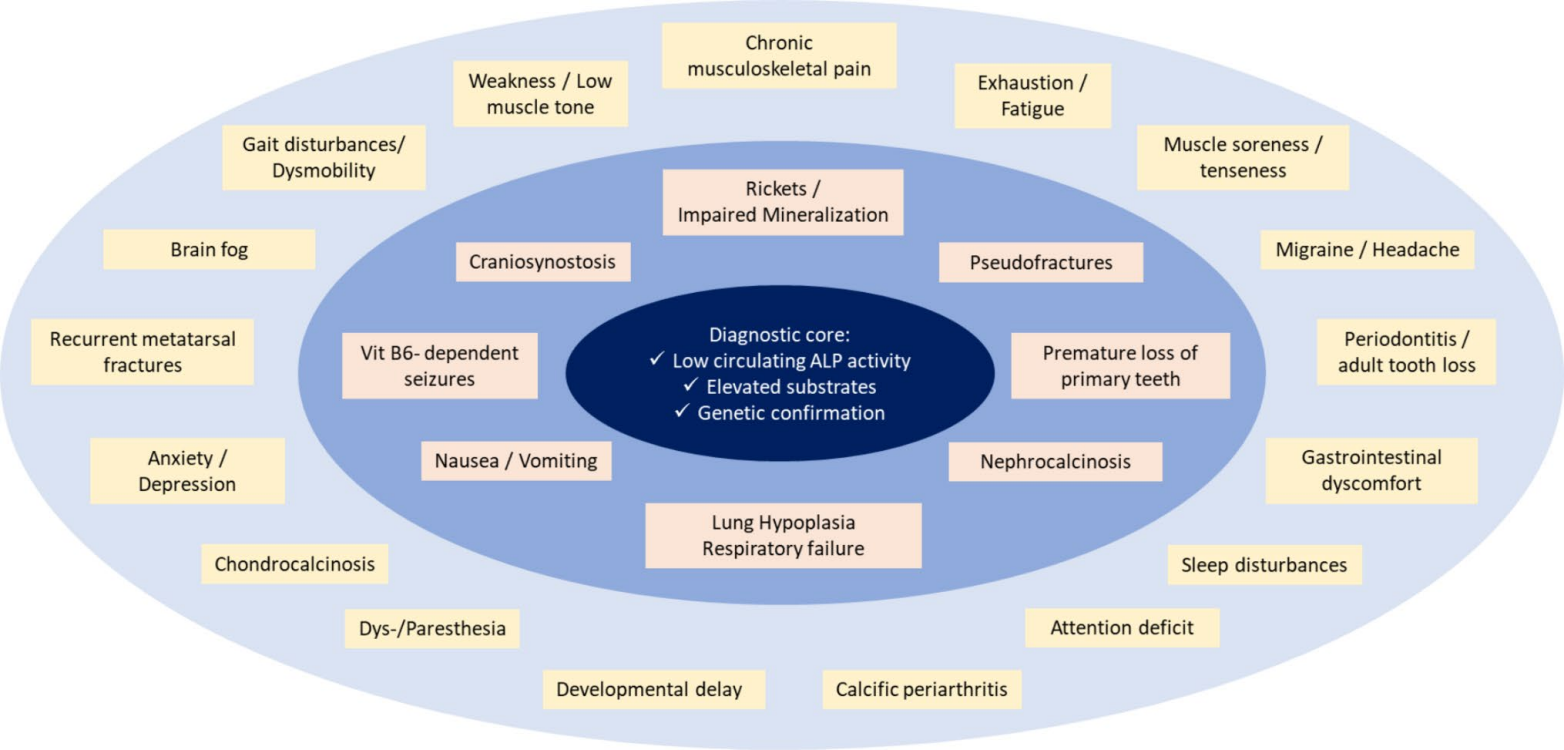
Graphic depiction of clinical signs and symptoms of Hypophosphatasia. Low circulating ALP activity, and elevated substrates due genetic ALPL variants reflect the pathophysiology of the disease and are thus considered the diagnostic core. The inner circle comprises canonical clinical manifestations that were unambiguously associated with severe HPP over decades, while the outer circle represents less specific manifestations

A high-level, age-independent suggestion to integrate canonical manifestations of the disease with less characteristic but nonetheless very common non-canonical signs and symptoms around an objective diagnostic core of genetics and biochemistry is provided in Fig. 2.

It is important to acknowledge that adults with canonical manifestations in early childhood may additionally experience such as non-canonical symptoms and it is increasingly recognized that well-established canonical manifestations and non-canonical signs and symptoms are not mutually exclusive.

**Fig. 3:**
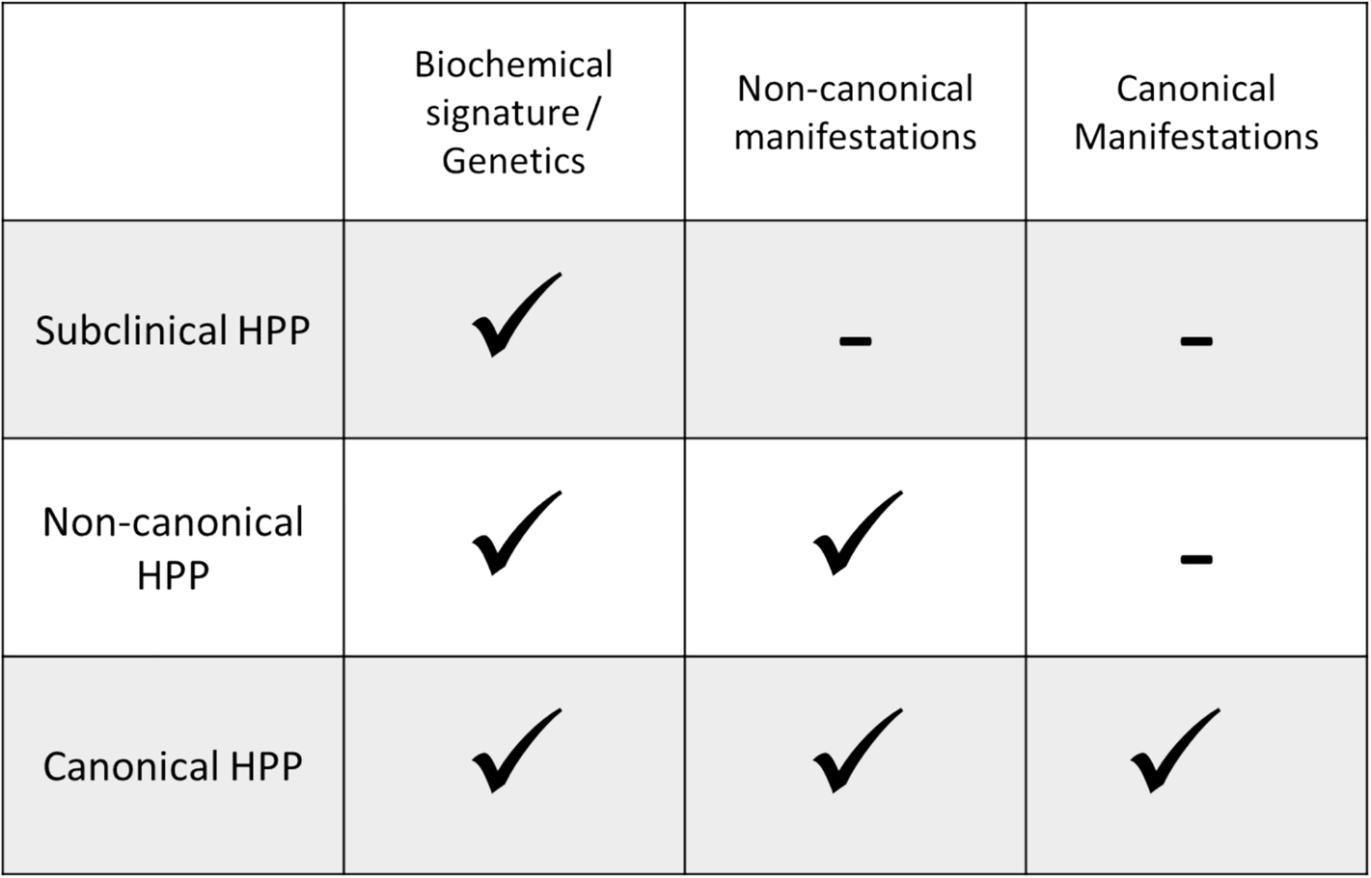
Hypophosphatasia assessment, differentiating “subclinical-HPP” with an ALPL Variant with or without a biochemical signature but no clinical manifestation from patients with additional non-canonical clinical manifestations. Patients with the fully evolved picture of HPP including and one or more “canonical” manifestations in most instances also experience non-canonical” signs and symptoms

In summary, hypophosphatasia must be seen as a lifelong, systemic condition. Considerations around diagnosis, nosology, and treatment should overcome limitations imposed by age, alleged onset and single organ or tissue manifestations like e.g., “odonto”-HPP. Figure 3 provides a high-level suggestion on how this could be integrated in clinical practice.

## Therapeutic Approaches in HPP

### Critical Care

Severe perinatal manifestations of HPP constitute a critical situation, demanding timely therapeutic interventions and intensive care in most instances. Specifically, these infants frequently experience respiratory failure and Vitamin B6-dependent seizures, both of which significantly impact survival and the overall course of the disease. They require ventilatory support and parenteral, pyridoxine hydrochloride [57]. However, timely start of enzyme replacement has turned out to be critical in that regard [47] and the availability of asfotase alfa has fundamentally changed the prognosis of these infants not only in terms of skeletal health and respiratory function but also has significantly improved overall prognosis and survival [58, 59].

**Fig. 4:**
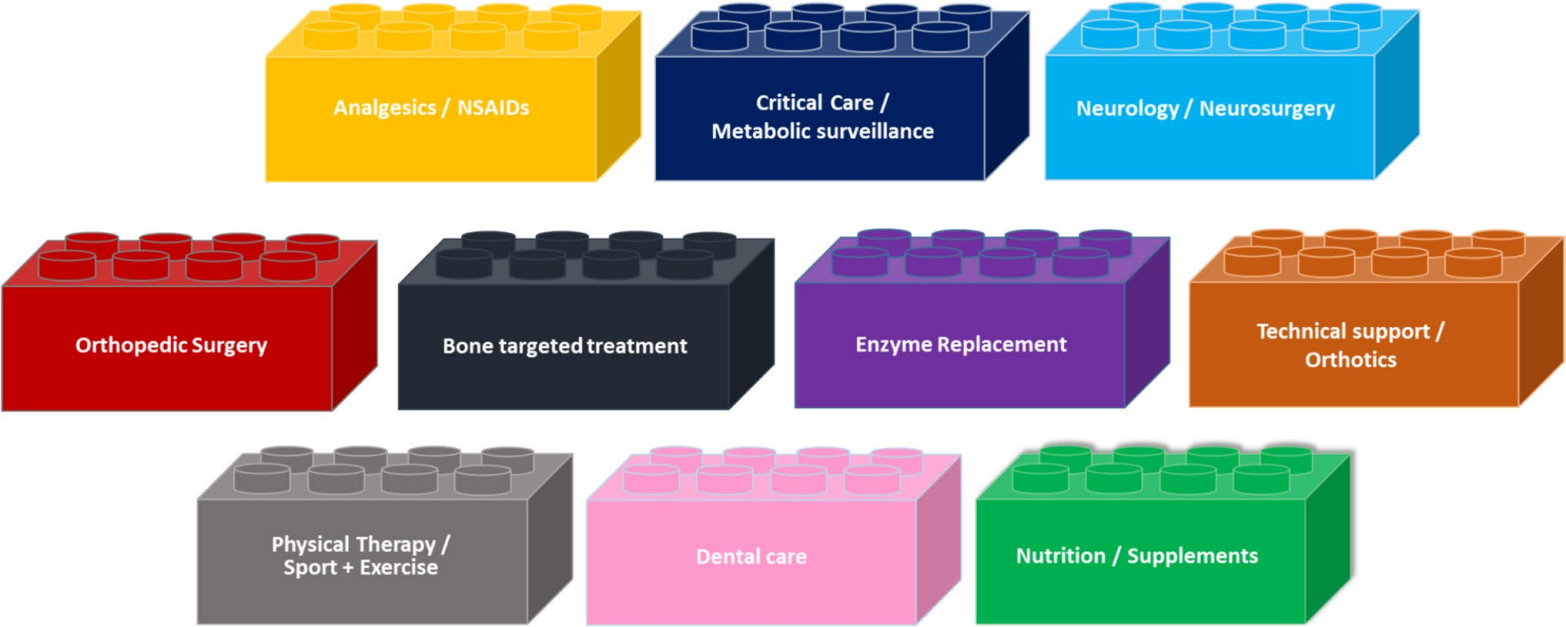
Components of multimodal care for HPP patients, depicted as building blocks to reflect the need for individualized composition of different modules depending on clinical manifestation and age to enable optimal personalized management

Craniosynostosis can become clinically apparent in children with HPP during their first years of life, leading not only to skull deformities but also bearing the risk of a critical increase in intracranial pressure and associated complications [32, 60]. Hence, regular clinical neurosurgical assessments and imaging plus fundoscopy to identify papilledema with appropriate imaging are vital to avoid missing potential need for surgical intervention, which can include cranial vault remodeling (Fig. 4).

### Enzyme Replacement Therapy

ERT has substantially changed the prognosis of severely affected children with HPP and markedly improved survival rates by enabling skeletal mineralization by lowering PPi levels and improving availability of dephosphorylated vitamin B6 be reducing PLP [58, 59]. Children on ERT show progressive healing of rickets with reduced skeletal deformities and improved motor function. While long-term effects of ERT are still being investigated, available evidence to date underscores a clearly positive risk–benefit ratio in children with early onset and severe clinical manifestation. While the vast majority of available experience with ERT concerns children, approval of asfotase alfa for long-term enzyme replacement therapy (ERT) in most regions of the world also covers patients with pediatric-onset of the disease, irrespective of their current age [61]. Efficacy has also been shown for pseudofracture healing and bone mineralization in adults with HPP [62–65]. However, not all patients respond equally to ERT, and its effectiveness may vary depending on additional, as yet unidentified individual patient factors. Furthermore, while bone-targeted ERT seems to consistently improve the underlying bone pathology in HPP, it may not fully alleviate extra-skeletal symptoms like pain, fatigue, and functional limitations [63, 64, 66].

Prospectively, ongoing research may fortunately expand the scope of treatment options for hypophosphatasia and various concepts are being developed. With efzimfotase alfa, a next-generation development of asfotase alfa [67] and Ilofotase alfa (NCT05890794), two novel compounds have passed phase I evaluation for HPP and further therapeutic modalities including small molecules, as well as gene-based and cell-based strategies are undergoing pre-clinical evaluations. Specifically, when it comes to the large group of patients with non-canonical and not life-threatening manifestations, larger scale placebo-controlled/cross-over studies, with less burdensome treatment applications would be warranted to substantiate the benefit for these patients.

### Analgesic Therapy

As children grow, analgesic treatment can have an important supportive role in managing pain and discomfort in children with HPP to facilitate physiologic development, particularly in those experiencing skeletal abnormalities and fractures. Naproxen, a nonsteroidal anti-inflammatory drug (NSAID), has been shown to yield positive effects in cases of pediatric HPP patients [32, 68, 69]. Considering the high prevalence of inflammatory pain in adults with HPP [27, 29, 30, 70] the use of NSAIDs for supportive care is even more common in this patient group. However, in the absence of specific evidence, pain management in adult HPP patients is largely based on personal experience. In that regard, NSAIDs and specifically ibuprofen as needed, but restricted to the lowest effective dose seem to have a favorable risk–benefit ratio while centrally acting analgesics, specifically opioids, are of limited use and have significant downsides.

### Bone-Targeted Treatment

While patients with HPP-related mineralization disorders and a canonical phenotype frequently exhibit particularly high lumbar spine BMD values [71] associated with HPP, less severely affected adult HPP patients may develop osteoporosis and a risk for osteoporotic fractures, by means pathophysiologically independent of an underlying ALPL variant. This specifically applies to biochemically milder affected patient, i.e., patients with a non-canonical phenotype [49, 72–74]. Clinical experience implies that this should be regarded as a coincidence of two skeletal conditions instead of considering osteoporosis an inherent consequence of HPP. Neither is HPP associated with an increased risk for major osteoporotic fractures of e.g., vertebrae or the metaphyseal parts of the hip and the humerus, nor is it causally associated with low BMD [75]. However, being mindful of the reduced remodeling activity in HPP [76] and the disease-specific accumulation of inorganic pyrophosphate, concerns regarding the use of bisphosphonates (BP) in such a constellation are justified. On the one hand, BP as pyrophosphate-analogs—specifically when used in large amounts or for a significant length of time, may similarly interfere with mineralization, given inherently elevated PPi. This has been well-described in the older non-amino bisphosphonates which had to be applied in large amounts and case series support the assumption of a potential detrimental effect on pseudofracture risk in HPP patients with inherently elevated PPi levels [50]. In addition, by reducing bone resorbing osteoclasts’ activity, BPs also limited their ensuing impact on recruitment and activation of osteoblasts, osteoblastic precursors and reversal cells thus suppress overall remodeling activity even further [50, 77, 78]. These concerns extend to the use of denosumab, as it too suppresses bone turnover and—along with that—ALP activity.

Conversely, the use of osteoanabolic therapeutics, specifically teriparatide and anti-sclerostin antibodies appears safe and feasible in HPP. While data regarding their efficacy are scarce, several case reports and a small phase II study with an anti-sclerostin antibody support their application to be safe and effective in terms of increasing BMD in HPP patients [79–81]. Still, larger studies to provide fracture data are missing and such treatments should only be initiated with a clearly defined treatment objective and a long-term strategy to maintain bone health beyond the first dosing cycle. Conversely, in patients with prevailing HPP on ERT, available data support that this will concurrently increase BMD [82].

### Physical Therapy, Sport, and Exercise

A state of physical fatigue and perceived exhaustion with generalized pain, weakness, and reduced exercise tolerance represent the most frequently reported, non-canonical manifestation of HPP, especially in adults [83]. Acquisition of strength and mobility in childhood, and maintenance of physical fitness in adulthood, are central determinants of patients' perceived burden of the disease and disease-related quality of life [84, 85]. Physiotherapy and exercise interventions under expert guidance, ideally combined with instructions for a home program are empirically promising and do not bear a significant risk or undesirable side effects [86]. Given the disproportionately frequent complaints about muscular tension and stiffness, particularly in the neck and shoulder girdle region, addition of relaxation techniques may be helpful, but data on specific techniques are lacking [87, 88].

From our personal experience, leisure and sports activities, diversified exercises involving several muscle groups and supporting their flexibility while avoiding overexertion appears preferable against tedious, monotonous, and specifically isometric workouts or overexertion, with all of the latter bearing the risk of prolonged pain and fatigue. This type of pain is what HPP patients refer to when talking about “exercise intolerance” and this is what jeopardizes patients’ motivation for sustainable activity. Available evidence supports the notion that ERT significantly facilitates motor development in children and may alleviate muscular symptoms, and thus facilitate physical training adults [63, 66]. However, the underlying molecular mechanisms still warrant further research to better understand individual variability of this effect. Overall, physical exercise interventions appear beneficial and are to be recommended for all patients irrespective of age, disease severity, and the indication for ERT but should be tailored according to personal preferences and tolerance levels.

### Orthotics

In both adult and pediatric HPP patients, orthotics can be supportive in managing the musculoskeletal manifestations associated with the condition. The spectrum of available devices ranges from simple insoles to complex, stabilizing or functional orthoses, and complex technical support. In some cases, off the shelf orthotics can be used, but in most cases custom orthotics will be a superior option [89]. Orthotics can provide external support but can also guide gradual alignment, not only to prevent deterioration but ideally also to progressively correct deformities during growth in children. Considering the prevalence of low muscular tone and deficient muscular stabilization in children with HPP posing them at risk for deformities such as plano-valgus deformity of the feet, genu valgum, and scoliosis, mindful orthopedic bracing can be helpful to prevent progression or even support normalization of such sequelae of the disease [90, 91]. In case of moderate deformities, cautious orthotic treatment may even be sufficient to maintain proper posture and mobility and overcome the need for surgical intervention. For adults, especially those with long-standing skeletal deformities or complications from childhood and/or orthopedic interventions, orthotic treatment can alleviate pain, improve mobility and function, and prevent further structural deterioration [92].

### Nutrition and Supplements

As with the general population, there are a lot of conflicting recommendations for nutritional counseling in HPP. However, evidence basis is limited with only a few well-defined situations that warrant dietary intervention. In severe, perinatal HPP at risk of vitamin B6-dependent seizures, parenteral pyridoxine hydrochloride therapy is indicated [57, 93] to mitigate the risk for Vitamin B6-dependent seizures since circulating PLP cannot cross the blood–brain barrier in the absence of ALP-mediated dephosphorylation. Furthermore, severely affected infants and young children with perpetual nausea and vomiting may require nutritional support and even PEG tube feeding to avoid nutritional deficits and ensure adequate growth and development. Considering the aspect of compromised integration of calcium and phosphate into the bone with the risk of elevated circulation levels and ectopic calcifications specifically in infants and without ERT, mineral metabolism, particularly vitamin D, PTH, calcium, and phosphorus should be monitored regularly to prevent hyperphosphatemia and hypercalcemia.

For adult patients, empirical recommendations frequently insinuate advantages of a low phosphate diet. However, scientific data to support this advice are lacking and our own work in that regard confirms the advantage of a balanced approach, avoiding both excess or insufficient dietary phosphate [12, 13]. Additionally, recent studies did not substantiate clinical benefits of increased dietary intake of zinc and magnesium, which are cofactors of the enzyme [12]. Supplementation with Vitamin D in patients with HPP does not deviate from established guidelines for the general population in both children and adults with HPP.

### Surgical Intervention

In cases with severe skeletal deformities, i.e., functionally relevant or even disabling deviations of the long bones, corrective surgery may be required. With thoughtful surveillance and timely planning, less invasive procedures with guided growth by transient hemiepiphysiodesis are frequently appropriate to address such issues [94, 95]. In more severe deformities, corrective surgeries can be required to effectively correct skeletal deformities. Such procedures should be performed by orthopedic surgeons familiar with the pathophysiology and the unique bone physiology of HPP.

Similarly, surgical stabilization of (pseudo-)fractures should be accomplished in a setting that is competent in coordinating appropriate pharmaceutical interventions with suitable surgical approaches, considering bone quality and the anticipated course of healing [50]. If surgical stabilization is required for complete fractures, symptomatic pseudofractures, and still asymptomatic but progressive pseudofractures, it is recommended to utilize load-sharing internal-fixation devices, such as rigid intramedullary nails [50, 96]. These devices provide structural support to the bone and facilitate proper healing. Given the risk of recurrence, it is important to avoid unnecessary removal of these implants. If removal becomes necessary due to complications or for other reasons, circumspect evaluation of the resulting mechanical situation and the need for adequate replacement must be assessed before in order to minimize the risk of additional trauma and instability over time in case of pseudofracture recurrence.

With progressive aging, osteoarthritis in the large, weight-bearing joints appears to be a critical factor limiting mobility and activity in HPP patients. It remains unclear to what extent this is related to the metabolic state itself or rather a consequence of prevalent deformities, mobility restraints, and altered biomechanics and mechanical loading. Either way, joint replacement surgery in HPP is technically feasible but should be accomplished with diligent preparation, integrating pharmacological preparation of the bone, adjusted selection of the utilized implant and fixation technique.

### Perspective

Much has been achieved regarding diagnosis and treatment for patients living with HPP but this does not obviate the need for further efforts to better understand the disease and further improve our current understanding of the disease. The above considerations around non-canonical manifestations of HPP surely require further substantiation, specifically regarding their frequency and the associated individual and also societal burden. Consequently, the need for a multidisciplinary management across the life span is important and depending on individual phenotypic presentation, this multidisciplinary team may involve pediatricians, endocrinologists, geneticists, orthopedic surgeons, dentists, and many other disciplines. Early recognition of clinical features, accurate diagnostic testing, and tailored therapeutic interventions will be essential for optimizing patient outcomes and improving long-term perspective.

Continued research efforts aimed at elucidating the pathophysiology of HPP, identifying novel therapeutic targets, and evaluating the long-term safety and efficacy of existing treatments are warranted to address the still largely unmet needs of so many individuals affected by this complex disorder.
